# Exploring the impact of naltrexone on the THBS1/eNOS/NO pathway in osteoporotic bile duct-ligated rats

**DOI:** 10.1038/s41598-023-50547-w

**Published:** 2024-01-02

**Authors:** Seyed Reza Hosseini-Fard, Shahroo Etemad-Moghadam, Mojgan Alaeddini, Ahmad Reza Dehpour, Solaleh Emamgholipour, Abolfazl Golestani

**Affiliations:** 1 Department of Clinical Biochemistry, School of Medicine, Tehran University of Medical Sciences, Tehran, Iran; 2 Dental Research Center, Dentistry Research Institute, Tehran University of Medical Sciences, Tehran, Iran; 3 Department of Pharmacology, School of Medicine, Tehran University of Medical Sciences, Tehran, Iran; 4 Experimental Medicine Research Center, Tehran University of Medical Sciences, Tehran, Iran

**Keywords:** Clinical pharmacology, Endocrine system and metabolic diseases

## Abstract

Hepatic osteodystrophy, a prevalent manifestation of metabolic bone disease, can arise in the context of chronic liver disease. The THBS1-eNOS-NO signaling pathway plays a pivotal role in the maturation of osteoclast precursors. This study aimed to investigate the impact of Naltrexone (NTX) on bone loss by examining the THBS1-eNOS-NO signaling pathways in bile duct ligated (BDL) rats. Male Wistar rats were randomly divided into five groups (n = 10 per group): control, sham-operated + normal saline, BDL + normal saline, sham-operated + NTX (10 mg/kg), and BDL + NTX. Parameters related to liver injury were measured at the study's conclusion, and Masson-trichrome staining was employed to evaluate collagen deposition in liver tissue. Bone THBS-1 and endothelial nitric oxide synthase (eNOS) expression levels were measured using real-time PCR, while the level of bone nitric oxide (NO) was assessed through a colorimetric assay. NTX treatment significantly attenuated the BDL-induced increase in circulating levels of liver enzymes and bilirubin. THBS-1 expression levels, elevated after BDL, were significantly suppressed following NTX administration in the BDL + NTX group. Despite no alterations in eNOS expression between groups, the bone NO level, significantly decreased in the BDL group, was significantly reduced by NTX in the BDL + NTX group. This study partly provides insights into the possible molecular mechanisms in BDL-induced osteoporosis and highlights the modulating effect of NTX on these pathways. Further research is needed to establish the impact of NTX on histomorphometric indexes.

## Introduction

Hepatic osteodystrophy (HO) is a commonly used term to describe the metabolic bone disease that develops in patients with chronic liver disease (CLD)^1^. The exact pathophysiological mechanism of HO remains unclear and is probably multifactorial^2^. Various factors are associated with HO and are considered potential risk factors for disease progression. These factors include, but are not limited to: the length and severity of existing liver disease, body mass index (BMI), age, genetic predisposition, inadequate nutrition or dietary insufficiencies, hormonal status, low bone mineral density, history of fragile fractures, accumulation of copper and iron, changes in vitamin levels, elevated bilirubin levels, and the effects of prescribed medications^3^. The incidence of osteoporosis in individuals with CLD is reported to range from 12 to 55%^4^. It is well-established that HO disrupts bone remodeling, a continuous and tightly coordinated process involving both bone resorption and formation^5^. Imbalances in bone remodeling lead to a reduction in bone quality, consequently resulting in pathological bone fractures^6^. Moreover, the primary mechanism driving osteoporosis in HO appears to be heightened bone resorption^7,8^. Osteoclasts (OCs) degrade bone by creating an acid compartment at their attachment site and secreting various proteases. Osteoclastogenesis, a complex and multistep process, involves the formation of mature OCs responsible for breaking down both the organic and inorganic components of formed bones^9^. The pathogenesis of HO is shaped by a broad spectrum of factors that can regulate the activity and differentiation of osteoclasts^1,10^. However, there remains considerable uncertainty regarding the role of proteins sequestered in the osteoid deposited by osteoblasts (OB) in the regulation of osteoclastogenesis^11^. The inhibition of the bone resorption activity of osteoclasts remains a high priority for most current osteoporosis therapies^12,13^. Hence, understanding the mechanisms of osteoclast activity regulation is of paramount importance to alleviate osteoporosis in the future.

Emerging strategies are under development to prevent, delay, or reverse bone loss. However, a crucial step in this endeavor is to comprehend the underlying molecular processes. Researchers are investigating diverse mechanisms that could potentially serve as targets for these treatments^3^. Some potential targets include alterations in vitamin D metabolism^3^, imbalances in TGF-β^3^ and BMP signaling^14^, changes in the expression and function of histone deacetylases^15,16^, and the role of sclerostin in regulating the RANKL-OPG system, which in turn influences bone remodeling. Understanding and targeting these mechanisms could lead to more effective treatments for bone loss^17,18^. Recent advances proposed an undeniable role of extracellular proteins in cell function regulation. Thrombospondin-1 (THBS-1) is a large glycoprotein that binds to a variety of receptors such as CD36, integrins, and CD47^19^, and thus regulates signaling pathways in OCs^20^. By modulating cellular adhesion, migration, and differentiation, THBS-1 engages in various processes such as fibrosis and regulation of angiogenesis^21–23^. THBS-1 mimetics and humanized antibodies have entered cancer treatment preclinical and clinical studies^24,25^. Hence, an in-depth understanding of the role of THBS-1 in other organs, including bones where THBS-1 and its receptor come into play in regulating OC activity, seems warranted. Kerr et al. in 2019 showed that the THBS-1/TGF-β1 axis regulates pre-metastatic niche formation and tumor-induced bone turnover. Targeting the platelet release of TSP-1 or TGF-β1 represents a potential method to interfere with the process of metastasis to bone^26^. THBS-1 is secreted by OBs and is present as a sequestered protein in osteoid and mineralized bone^27^. It binds to its two cell-associated receptors CD36 and CD47, and by inhibiting endothelial nitric oxide synthase (eNOS) activity and nitric oxide (NO) signaling, promotes osteoclast differentiation^22^. There is evidence that THBS-1 deficient mice have increased cortical bone size and thickness. Moreover, CD47^-/-^ mice demonstrate defects in bone resorption^22^. Based on other surveys, both CD36 and CD47 are required for OC differentiation and maturation and more interestingly, anti-THBS-1 antibodies completely suppress the differentiation of these cells. However, L-NAME treatment has been shown to rescue this inhibitory effect^20^. Based on the above-mentioned information, it appears that THBS-1 and its receptors may be pivotal players in HO pathogenesis.

Naltrexone (NTX) is an FDA-approved opioid receptor (OR) modulator with a high affinity for μ-ORs and a low affinity for κ/δ-ORs^28,29^. A study has demonstrated an increase in the levels of endogenous opioids in individuals with liver cirrhosi^30^. Also, NTX has been found to protect against liver cirrhosis and its subsequent complications^31^. Ebrahimkhani et al. found a positive correlation between δ-OR expression levels and the severity of liver cirrhosis. This was evidenced by increased procollagen-I and tissue inhibitor matrix metalloproteinase-1 expression in activated rat hepatic stellate cells treated with δ-OR agonists^30^. Another study demonstrated that NTX ameliorates liver steatosis by reducing ER stress indicators^32^. In 2019, Tanaka et al. demonstrated that NTX enhances bone mass by increasing osteoblast numbers through antagonizing OGFR signaling. Their findings suggest that blocking opioid receptors holds promise as a therapeutic approach for osteoporosis^33^. In a 2020 study, it was demonstrated that NTX interacts with opioid receptors to inhibit the TLR4/NF-κB axes. This interaction plays a role in regulating the body's immune response, reducing the formation of osteoclasts—cells responsible for breaking down bone tissue. Consequently, NTX contributes to alleviating inflammation and preventing the erosion of both articular cartilage and bone tissue^34^. In addition, our previous investigations have shown that NTX could reduce bone loss in bile-duct-ligation (BDL) rats by modulating OPG/RANKL and TRAIL/sclerostin levels^35,36^. Here, we first evaluate bone levels of THBS-1, eNOS, and NO levels in BDL-induced cirrhotic rats. Considering the effect of endogenous opioids on bone loss, we subsequently intended to investigate the possible effect of NTX on the above-mentioned factors to uncover the potential role of the opioid system on hepatic osteodystrophy.

## Results

### The effect of NTX on BDL-induced hepatic collagen deposition, radiographic bone density, and bone histomorphometry

**Figure 1:**
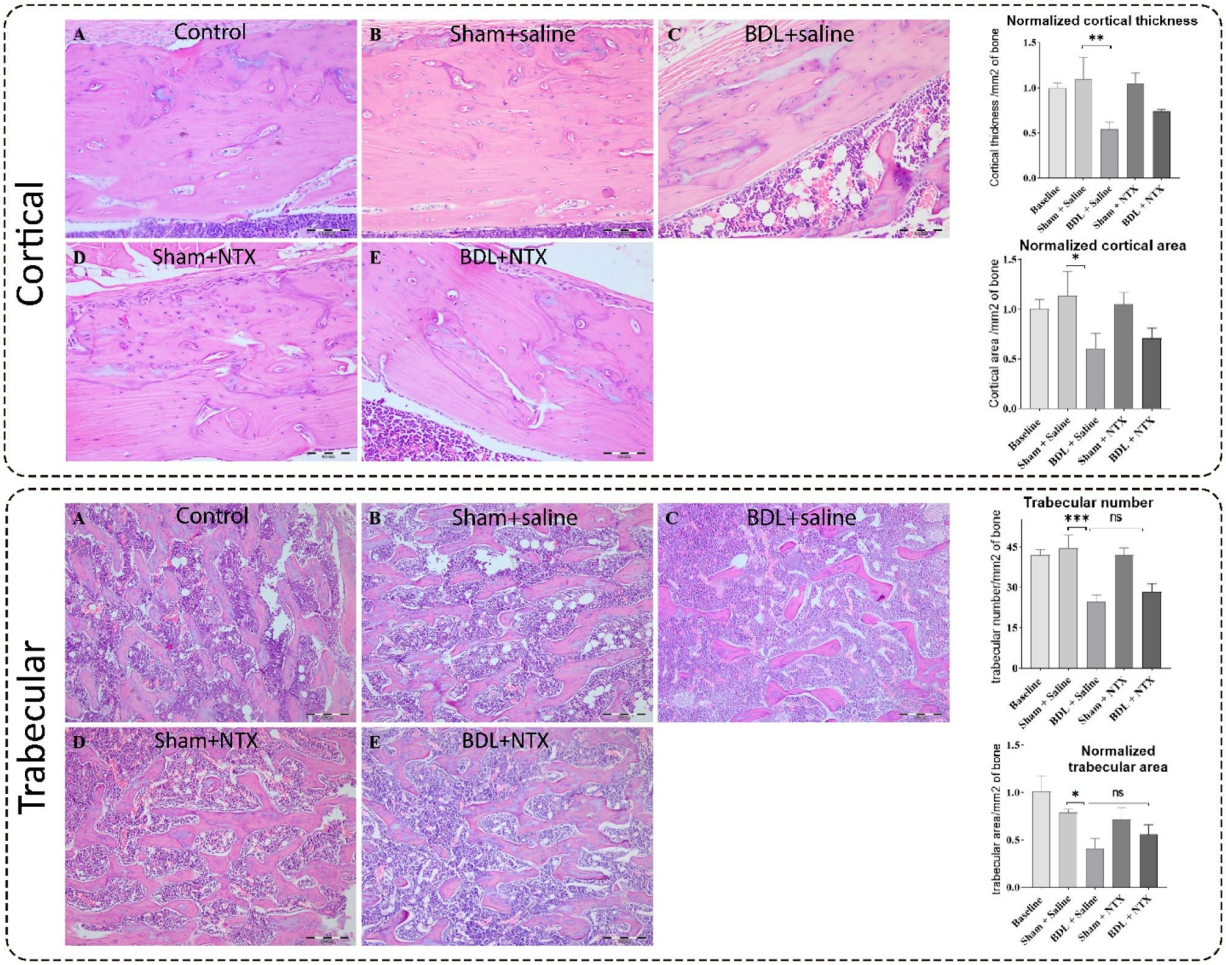
Representative hematoxylin–eosin-stained sections of rat tibia demonstrating cortical thickness/area and trabecular thickness/number in all study groups including controls, sham-operated and bile duct ligation (BDL) animals injected with saline, and sham-operated and BDL rats receiving naltrexone (NTX) (scale bar for all images: 0.1mm). Bar graphs show the comparison of treatment groups with controls (baseline) with *, ***, and NS representing < 0.05, < 0.0001, and no significant differences, respectively.

**Figure 2:**
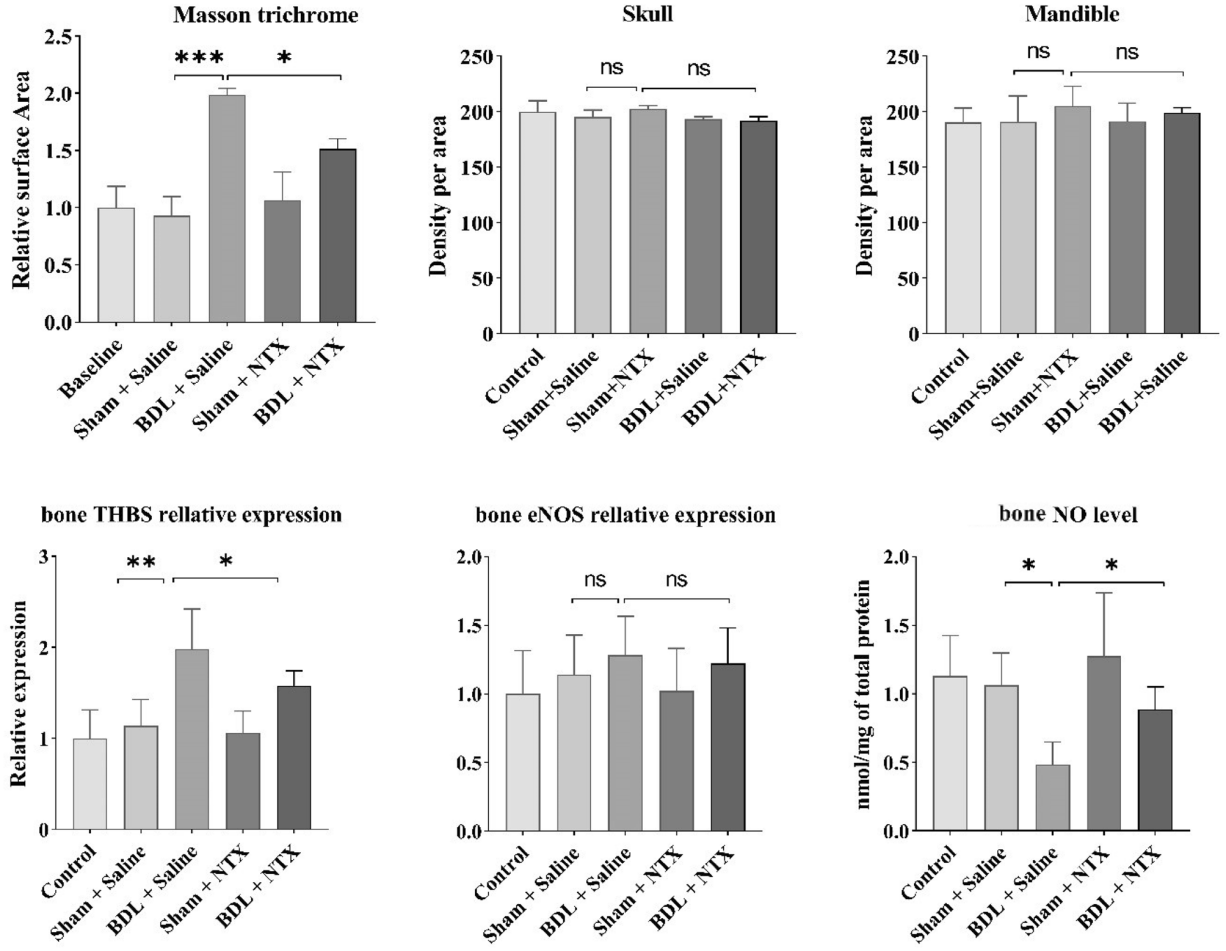
Optical density of the skull and mandible reference points in addition to the mRNA expression levels of thrombospondin-1 (THBS-1), endothelial nitric oxide synthase (eNOS) and nitric oxide (NO) among the study groups. Bar graphs show the comparison of treatment groups with controls (baseline) with *, **, and NS representing < 0.05, < 0.001, and no significant differences, respectively.

The BDL surgery resulted in a significant increase in collagen deposition within the BDL group (p = 0.0004). Notably, the administration of NTX demonstrated a significant decrease in collagen deposition in the BDL + NTX group compared to the BDL group. As illustrated in Fig. 1, BLD had a substantial impact on histomorphometric indexes, including a decrease in cortical bone thickness (P value = 0.0026), cortical bone area (p value = 0.02), trabecular bone thickness (P value = 0.018), and trabecular bone number (P value < 0.001) when compared to sham-operated animals receiving normal saline. Importantly, NTX treatment did not induce significant changes in the studied variables, as evidenced by the comparisons between the BDL + NTX and BDL + saline groups (Fig. 1). Additionally, there were no significant differences in radiographic bone density among the groups (Fig. 2).

### The effects of NTX on BDL-induced alterations in THBS-1, eNOS, and NO levels

The mRNA expression of THBS-1 in osseous tissue exhibited a significant increase in the BDL group compared to the controls (p value = 0.0025), and this elevation was significantly attenuated with NTX treatment (p-value = 0.012). Notably, there was no statistically significant difference in eNOS transcript levels among the study groups. Furthermore, the assessment of NO levels in bones revealed a noteworthy decrease in the BDL group compared to the sham-operated animals (P = 0.012), with a significant reversal observed following NTX administration (P = 0.039). These findings underscore the regulatory impact of NTX on key molecular components associated with bone metabolism and suggest a potential therapeutic avenue for mitigating hepatic osteodystrophy.

## Discussion

Liver cirrhosis and the consequent development of osteoporosis have become prevalent worldwide. NTX is gaining attention as a promising treatment strategy for addressing this disease.^31,35–37^. Consistent with this perspective, our observations affirm the dual efficacy of NTX, manifesting both anti-cirrhotic and bone-modulating effects. This is evidenced by the reduction in collagen deposition and the regulation of the THBS1-eNOS-NO axis in BDL rats.

The BDL rat model was successfully implemented in the current study, as indicated by the gradual yellow discoloration of the ears and urine, along with histopathological alterations in the liver. According to Dulundu et al.^38^ and Lee et al.^39^, BDL surgery elevates collagen deposition in hepatic tissues. Our previous study has shown that BDL surgery could increase oxidative stress in the liver of BDL rats. Moreover, we demonstrated that increased levels of thrombospondin-1 and NOX-1, as well as decreased levels of NRF-2 in the liver, may be important in the aggravation of oxidative stress in the liver of BDL rats^31^. Another study has proposed that a decreased level of glutathione (GSH) and an increased level of glutathione disulfide (GSSG) could affect the redox state of the liver and induce oxidative stress^30^. Another study proposed that hepatic oxidative stress might trigger the activation of hepatic stellate cells (HSCs), consequently inducing the synthesis and release of collagen^40^. Therefore, our observations indicating elevated collagen levels in BDL rats might be linked to reactive oxygen species (ROS)-mediated hepatic stellate cell (HSC) activation. Notably, the administration of NTX to BDL rats resulted in a significant reduction in collagen content in hepatic tissues. Consistent with our prior study, this effect may be attributed to the decrease in THBS-1 and NOX-1 levels, coupled with an increase in NRF-2, all induced by NTX^31^.

According to our findings, BDL induced a noteworthy decrease in tibial cortical thickness/area and trabecular thickness/number. Nevertheless, NTX injection did not lead to a statistically significant increase in these parameters. Nevertheless, additional studies have shown notable reductions in one or a combination of these indices in rats undergoing BDL-induced cholestasis^41–43^. Decreased levels of these parameters may indicate the successful execution of our BDL surgery. In 2015, Amend et al. proposed that the knockdown of THBS-1 in a mouse model led to an augmentation in cortical size and thickness of the tibia bone. They attributed this outcome to a reduction in THBS-1-mediated osteoclastogenesis^44^. Consistent with this investigation, our results demonstrated an elevation in THBS-1 levels in the bones of BDL rats. Therefore, the observed decrease in the aforementioned parameters may be associated with the increased level of THBS-1. Our study also revealed that NTX has a partial, albeit non-significant, effect on enhancing these parameters, concurrently decreasing the expression level of THBS-1. Similarly, previous research has demonstrated an improvement in histomorphometric variables in BDL rats following NTX administration, as evidenced by enhanced cortical area levels^41^. Our previous study has also shown that NTX mitigates osteoporosis-related markers by reducing the levels of TRAIL, adiponectin, and sclerostin in BDL-induced osteoporosis^36^. The insignificant change observed in the current investigation might be attributed to the possibility that specific effects could require more time to manifest and become observable in hard tissues at the microscopic level. Additionally, the relatively shorter duration of NTX injections in our study, as compared to the more extended periods employed in previous research, might have contributed to this non-significant change.

Subsequently, we examined the changes in bone THBS-1, eNOS, and NO levels. THBS-1 is a pivotal extracellular matrix (ECM) glycoprotein, and existing data suggest its potential role in regulating bone cell function. In this context, a study has proposed that osteoblasts may synthesize and release THBS-1 into the bone ECM. Additionally, the study indicated that THBS-1 enhances osteoclast maturation by reducing NO. They validated the role of NO signaling in osteoclast maturation through L-NAME treatment in THBS-1−/− mice^44^.

Concurrently, we noted that BDL resulted in the overexpression of THBS-1 compared to the corresponding sham-operated group. Amend et al. demonstrated increased bone mass and cortical thickness in THBS1-/- mice, suggesting that osteoblasts may synthesize and release THBS-1 into the bone extracellular matrix (ECM). Their study also indicated that THBS-1 promotes osteoclast maturation by reducing NO. Additionally, they emphasized the role of NO signaling in osteoclast maturation through L-NAME treatment in THBS-1−/− mice^44^.

Nevertheless, it has been demonstrated that THBS-1 knockdown can lead to a decrease in bone quality, as evidenced by reduced bone resistance to bending. Consequently, it has been suggested that THBS-1 may exert a double-edged sword effect on bone homeostasis. While THBS-1 silencing has been shown to decrease osteoclastogenesis, it has been proposed that, as an extracellular matrix (ECM) glycoprotein, the reduction of THBS-1 may negatively impact bone quality, increasing the risk of fractures^44^.

Another study has demonstrated the significance of THBS-1 in osteoclast formation, highlighting that the THBS1-CD36-CD47 pathway is influenced by changes in NO levels. This research has emphasized that suppressing CD36 or CD47 mitigates osteoclast formation, thereby inhibiting PTH-induced hypercalcemia in mouse models^20^.

Given the crucial role of NO signaling in osteoclastogenesis, our examination of eNOS mRNA levels revealed comparable expression across the groups. However, in contrast, we observed reduced NO levels following BDL surgery, which significantly improved after NTX treatment. This inconsistency could be elucidated by the elevated THBS-1 levels in the BDL group, which may inhibit eNOS activity through cell surface receptors. Additionally, another study has illustrated that THBS-1 could also decrease the expression level of inducible nitric oxide synthase (iNOS) in osteoclasts^44^, Hence, it can be speculated that the decreased level of NO in bone tissue may be a result of reduced NO production following BDL surgery. This effect was reversed by the NTX administration, which led to an increase in NO levels.

## Conclusion

The current study suggests that NTX may partly exert a positive effect on osseous tissues at the molecular level. This might be attributable in part to its reduction of THBS-1 mRNA and the induction of NO production. Our findings may partly provide fresh insights into the molecular mechanisms involved in the therapeutic efficacy of NTX in BDL-induced osteoporosis. However, further studies are warranted to establish this concept. Additionally, it is important to highlight that a study investigating the potential effects of an extended treatment period of NTX, surpassing 4 weeks, on the measured parameters could be warranted to better understand NTX efficacy.

## Methods

### Animals and reagents

The Department of Pharmacology, Tehran University of Medical Science (TUMS) provided all animals used in this study. We purchased NTX from Sigma (St Louis, USA), and ketamine hydrochloride and xylazine hydrochloride from Bremer Pharma (Warburg, Germany). The RNA extraction- and cDNA synthesis- kits were obtained from GeneAll (Seoul, Republic of Korea) and Takara (Shiga, Japan), respectively. We purchased the primers from Metabion (Steinkirchen, Germany) and the NO assay kit from Navand Salamat (Orumieh, Iran).

### Ethical approval

All experiments in animals were performed in compliance with the ARRIVE guidelines (https://arriveguidelines.org) and the relevant guidelines and regulations of the Institutional Animal Care and Use Committee (IACUC) of the Tehran University of Medical Sciences. The study was approved by the Ethical Committee of the Tehran University of Medical Sciences (IR.TUMS.MEDICINE.REC.1399.763).

### Experimental procedures

A total of 50 male Wistar rats were accommodated in a pathogen-free environment, with five rats per cage. The facility maintained controlled temperature and humidity levels, with the rats having ad libitum access to food and water, under a 12-h light and 12-h dark cycle. The rats were randomly allocated into five groups, each consisting of 10 rats: controls, sham-operated + normal saline, BDL + normal saline, sham-operated + NTX (10 mg/kg NTX), and BDL + NTX (10 mg/kg NTX).

To induce hepatic fibrosis, BDL was performed in the relevant groups after a 12-h fast, following established procedures. Briefly, the rats were fully anesthetized using 50 mg/kg ketamine HCL and 10 mg/kg xylazine HCL. The common bile duct (CBD) was precisely isolated through a 2-cm abdominal incision, followed by double-ligation and dissection. Sham-surgery groups underwent laparotomy with common bile duct exposure but no ligation, while the control group remained untreated. Both sham-operated and BDL rats received daily intravenous injections of either normal saline or NTX (10 mg/kg) for 4 weeks^35,36^.

At the end of the experiment, all rats were euthanized, and whole blood samples (5 ml) were collected in tubes containing an anticoagulant (10 IU/ml sodium heparin). The samples were then centrifuged at 2500 × *g* for 5 min at 4 °C, and the resulting plasma was frozen at − 80 °C for subsequent analysis. Liver tissues, left tibiae, and femur samples were promptly isolated and immersed in 10% neutral buffered formalin (NBF) for future use. The right tibiae were isolated and immediately frozen at − 80 °C for later analysis^31^.

All procedures were conducted under full anesthesia, as confirmed by the disappearance of corneal and pain reflexes. The survival rate following BDL surgery was 70%.

It's noteworthy that three mice from each group underwent assessments for liver injury, bone histomorphometric analysis, body density, and nitric oxide measurement. Additionally, five mice from each group were used for mRNA expression measurements.

### Liver injury assessment

For the investigation of BDL-induced liver injury, the collected tissues were immersed in 10% neutral buffered formalin (NBF) at room temperature. Following fixation, the tissues were processed, paraffin-embedded, sectioned at a thickness of 4–5 μm, mounted, and subsequently stained with Masson trichrome. Two independent pathologists analyzed the samples using a light microscope (Olympus CX31, Japan) and ImageJ software (version 1.51, NIH). The relative surface area of collagen deposition was quantified. Any discrepancies in interpretation were resolved through discussion between the pathologists.

### Bone histomorphometric analysis

All left tibiae were fixed in 10% neutral buffered formalin (NBF), subjected to decalcification in 14% ethylenediaminetetraacetic acid (EDTA), dehydrated through incremental ethanol concentrations, embedded in paraffin, sectioned (4–5 μm), and underwent routine processing for hematoxylin and eosin (H&E) staining. As previously described^41^, measurements were conducted at a distance of 195 μm from the epiphyseal growth plate using a light microscope (BX51, Olympus) equipped with an Olympus DP25 camera and DP2-BSW analysis software. Cortical bone thickness, cortical bone area, trabecular bone thickness, and trabecular bone number were evaluated by two blinded pathologists. Any discrepancies were resolved through discussion.

### Bone density assessment

It is well-established that optical density (OD) is inversely correlated with bone mineral density and has been extensively employed to assess bone-related conditions resulting from various diseases. On day 28, lateral cephalometric radiographs were captured from all rats using a custom-made cephalogram with precise positioning and projection control, as previously described^41^. The exposure conditions involved 10 mA, 0.25 s, and 50 kVp (peak kilovoltage). Subsequently, the radiographs were processed and analyzed using ImageJ software (NIH) to evaluate bone mineral density. OD readings were taken 1 mm beneath the most posterior point of the cranium and at the intersection between the Gonion to Menton (GO-Mn) line vertical to the Go-Mn line through Gnathion in the skulls and mandibles of the animals.

### Real-time polymerase chain reaction

The mRNA levels of THBS-1 and eNOS were assessed in the right femur samples through real-time PCR. Total RNA extraction from the right femur was carried out using a GeneAll kit following the manufacturer's instructions. RNA purity was determined by measuring the absorbance ratio at OD260/OD280 using spectrophotometry (Thermo Scientific NanoDrop).

Complementary DNA (cDNA) synthesis was conducted with the PrimeScrip™ RT Reagent Kit, and primer design (Table 1) was verified using Oligo 7 software and primer-BLAST. Optimal primer-annealing temperature was determined through gradient PCR and 1.5% agarose gel electrophoresis to ensure the presence of correctly sized fragments.

**Table 1:** Primer sequences used for Real-Time PCR.

	F Primer	R Primer	Product length (nt)
THBS-1	5´-CTCTCCTGTGACGAACTATCC-3´	5´-CTCTGTTCTCTTCCGTCACT-3´	106
eNOS	5´-GTATTTGATGCTCGGGACTG-3´	5´-TGATGGCTGAACGAAGATTG-3´	103
Beta-actin	5´-GCCAACCGTGAAAAGATGAC-3´	5´-AGCGCGTAACCCTCATAGAT-3´	173

Real-time PCR conditions included an initial denaturation at 95 °C for 5 min, followed by 40 cycles at 95 °C for 15 s, annealing at 62 °C for 30 s, and extension at 72 °C for 30 s, using Step-one plus (ABI, USA). Relative gene expression was calculated using the 2^−ΔΔCt^ formula. All data were normalized to the reference gene (β-actin), as well as the control group.

### Nitric oxide measurement

NO levels were measured on the right rat femurs using commercial kits. In brief, following bone homogenization, 20 mg of bone was combined with 200 µl of cold PBS, subjected to centrifugation at 13,000 *g* for 10 min at 4 °C, and the resulting supernatant was collected for protein quantification. Subsequent preparation steps were undertaken, culminating in the measurement of NO levels in bone samples at 570 nm using photometry.

### Statistical analysis

All data were analyzed by one-way ANOVA with Tukey posthoc analysis using GraphPad Prism v.8. Significant difference was set at < 0.05, and data were shown as mean ± standard deviation.

## Data Availability

The data that support the findings of this study are available from corresponding authors.
